# WIPI1, BAG1, and PEX3 Autophagy-Related Genes Are Relevant Melanoma Markers

**DOI:** 10.1155/2018/1471682

**Published:** 2018-12-02

**Authors:** Daniela D'Arcangelo, Claudia Giampietri, Mario Muscio, Francesca Scatozza, Francesco Facchiano, Antonio Facchiano

**Affiliations:** ^1^Istituto Dermopatico dell'Immacolata, IDI-IRCCS, via Monti di Creta 104, 00167 Rome, Italy; ^2^Department of Anatomical, Histological, Forensic Medicine and Orthopedic Sciences, Sapienza University, Rome, Italy; ^3^Department of Oncology and Molecular Medicine, Istituto Superiore di Sanità, ISS, viale Regina Elena 299, 00161 Rome, Italy

## Abstract

ROS and oxidative stress may promote autophagy; on the other hand, autophagy may help reduce oxidative damages. According to the known interplay of ROS, autophagy, and melanoma onset, we hypothesized that autophagy-related genes (ARGs) may represent useful melanoma biomarkers. We therefore analyzed the gene and protein expression of 222 ARGs in human melanoma samples, from 5 independent expression databases (overall 572 patients). Gene expression was first evaluated in the GEO database. Forty-two genes showed extremely high ability to discriminate melanoma from nevi (63 samples) according to ROC (AUC ≥ 0.85) and Mann-Whitney (*p* < 0.0001) analyses. The 9 genes never related to melanoma before were then in silico validated in the IST online database. BAG1, CHMP2B, PEX3, and WIPI1 confirmed a strong differential gene expression, in 355 samples. A second-round validation performed on the Human Protein Atlas database showed strong differential protein expression for BAG1, PEX3, and WIPI1 in melanoma *vs* control samples, according to the image analysis of 80 human histological sections. WIPI1 gene expression also showed a significant prognostic value (*p* < 0.0001) according to 102 melanoma patients' survival data. We finally addressed in Oncomine database whether WIPI1 overexpression is melanoma-specific. Within more than 20 cancer types, the most relevant WIPI1 expression change (*p* = 0.00002; fold change = 3.1) was observed in melanoma. Molecular/functional relationships of the investigated molecules with melanoma and their molecular/functional network were analyzed via Chilibot software, STRING analysis, and gene ontology enrichment analysis. We conclude that WIPI1 (AUC = 0.99), BAG1 (AUC = 1), and PEX3 (AUC = 0.93) are relevant novel melanoma markers at both gene and protein levels.

## 1. Introduction

Cutaneous melanoma is the most aggressive skin cancer; it accounts for less than 5% of all skin cancers but causes about 80% of skin cancer-related mortality. Effective diagnostic markers are therefore needed to reduce melanoma-related mortality. Melanoma pathogenesis is usually related to the skin phototype, and risk factors include environmental factors, lifestyles such as intermittent excessive UV exposure and sunburns, and genetic predisposition. Excessive UV exposure results in DNA damage and gene mutations, mostly on BRAF, in almost half of melanoma cases, leaving the remaining half without a specific genetic marker. Despite that melanoma genetics is under extensive investigation [1–4], a melanoma-specific genetic profile has not been identified yet, and early markers of melanoma onset are still unknown. While S100B and LDH are recognized as clinically reliable prognostic markers in late stages, LDH is the only marker accepted by NCCN and AJCC current melanoma guidelines for TNM scoring and clinical staging. Therefore, clinically relevant diagnostic early biomarkers are still to be identified. We [5, 6] and others identified mechanisms affecting melanoma onset, including immune surveillance control [7], IL8/bcl-XL axis [8], aberrant expression of kallikrein-related peptidase 7 [9], fatty acid oxidation [10], lipid droplet accumulation [6], and PDGF-R*α* expression in [11, 12].

The role autophagy plays in physio-pathological conditions is under intense investigation. It has both protective and detrimental effects, depending on the conditions [13], including pro- or antitumor effects [14–17]. As an example, chaperone-mediated autophagy degrades different substrates, with both cancer-suppressor and cancer-promoting activity [18]. Autophagy is considered as tumor-suppressive in melanoma early stages and tumor-promoting in melanoma later stages [19]. SQSTM1 and AMBRA1 are emerging as prognostic markers for early-stage melanoma [18, 20]. Further, endoplasmic reticulum stress-induced unfolded proteins [21, 22] appear to mediate the resistance to antimelanoma therapies, and removal dysfunctional/aged mitochondria, known as “mitophagy,” may play a role in melanoma setup and progression [23].

All such studies support a key role of autophagy-related genes (here referred to as ARGs) in melanoma setup. We therefore hypothesized that ARGs may represent suitable melanoma markers and investigated for the first time the expression levels of 222 ARGs in several melanoma samples. Several hundred human melanoma samples from 4 different and independent public databases in 4 consecutive validation stages were analyzed.

## 2. Materials and Methods

ARGs were from HADb, Human Autophagy Database, at http://www.autophagy.lu/clustering/, from the Luxemburg Institute of Health, including 222 genes at the date of March 2017 (Supplementary Table 1). Expression in a total of 572 human samples was investigated from 5 different public datasets, via 1 screening phase + 3 consecutive validations phases.

### 2.1. ARG Expression from the GEO Database: Melanoma *vs* Nevi Samples

Expression of 222 ARGs was evaluated in the melanoma GDS1375 dataset, from the GEO database (https://www.ncbi.nlm.nih.gov/gds/). This human dataset reports expression data from the most numerous collections of melanoma and nevi samples, available within GEO. It reports the actual expression values; calculations such as mean, Mann-Whitney test, and ROC analysis were then carried out on 63 samples (45 melanoma patients *vs* 18 nevi patients). ROC analysis, the most accepted method in binary tests, was used to measure how effective the expression level of any given gene to discriminate healthy from melanoma biopsies is. The computed area under the curve (AUC) value ranges from 0.5 to 1, indicating 50% to 100% discrimination ability. Sensitivity and specificity were selected from the expression levels showing the best sensitivity-specificity combination.

### 2.2. Selection of Genes Not Known to Have a Relation with Melanoma

Genes selected from the GEO analysis, having AUC ≥ 0.85, were then searched in Pubmed for any co-occurrence with melanoma, at the date of April 19, 2018. Search in “ALL fields” was carried out to minimize false-negative results. Genes showing no co-occurrence with melanoma were considered novel in the melanoma field and were selected for the following validation steps.

### 2.3. First-Round Validation of Gene Expression Data in the IST Online Database

The first-round validation step was carried out by analyzing expression levels of the selected genes in the independent IST Online database (http://ist.medisapiens.com/), having 208 melanoma samples and 147 healthy skin samples. Differently from the GEO database, IST Online does not show actual numbers; rather, it expresses data as scatter plots. Therefore, scatter plots were obtained according to a previously reported procedure [11]. Genes showing expression levels in melanoma strongly different to controls were then selected for the following validation steps.

### 2.4. Second-Round Validation: Protein Expression in the Human Protein Atlas Database

The 4 genes identified and validated within the previous phases were then analyzed at the protein expression level in Human Protein Atlas (https://www.proteinatlas.org/). Eighty histological sections (47 cutaneous melanoma and 33 healthy skin controls) were retrieved and transformed in grayscale by using the GIMP image editor (version 2.8) (GNU Image Manipulation Program; https://www.gimp.org) according to the following steps:

1. Image selection
2. Background removal by converting the white background to transparent
3. RGB image color conversion to grayscale
4. Histograms, mean, pixel numbers, median, and standard deviation obtained from the image menu: Colors → Information → Histogram

### 2.5. Chilibot Analysis

Chilibot analysis was then performed (http://www.chilibot.net) [24]. Chilibot identifies known relationships within the user-defined terms, by looking at their coexistence in the same sentence within Pubmed abstracts, identifying much closer relations as compared to a plain Pubmed search. Chilibot then associates same-sentence co-occurrence to stimulatory or inhibitory or noninteractive relationships. A “two-list analysis” was carried out, the first list containing the term “melanoma” and the second list containing the 42 ARG gene list of Table 1; the “advanced options” button was turned on, to account for all known synonyms of the given terms and minimize false-negative findings.

### 2.6. Third-Round Validation: Investigating the Specificity of WIPI1 Overexpression

Cancer types showing the most relevant WIPI1 upregulation were identified on Oncomine database (http://www.oncomine.com). WIPI1 expression in a “cancer *vs* normal” analysis was investigated in 170 independent human datasets from more than 20 different cancer types, onto 31,931 human biopsy samples; a list was obtained of cancer types and relevant datasets where WIPI1 is significantly over- or underexpressed.

A “cancer *vs* cancer analysis” was also carried out, with filter “Dataset type: Cell line panel datasets,” to address expression in all cancer lines available. In this case, 26 different datasets with 7410 samples from 18 different cancer types were analyzed.

The Oncomine search engine applies a combination of threshold values, namely, *p* value, fold change *vs* controls, and gene rank (rank of that gene in that dataset, based on its *p* value, indicating how many other genes in the analysis are more or less significant). Very strict thresholds were imposed, namely, *p* ≤ 0.0001, fold change ≥ 3, and gene rank top 5%. An additional validation of normal vs melanoma expression data was achieved by analyzing an additional dataset in the GEO database (namely, GSE 15605 containing 74 samples: 16 normal, 46 primary, and 12 metastatic-melanoma) and by accessing the Expression Atlas database at EMBL-EBI available at (https://www.ebi.ac.uk/gxa/home).

### 2.7. Network Analysis and Functional Grouping Analysis

Additional analyses were carried out to investigate the functional grouping and the structural/functional network of the identified genes. To this aim, the enrichment analysis was performed onto the 42 genes reported in Table 1, at the link http://www.geneontology.org/page/go-enrichment-analysis. It is a Gene Ontology tool that identifies GO terms over- or underrepresented within the GO annotations of the given gene list. In addition, the STRING analysis at https://string-db.org/ was performed to identify a functional network of the three main hits identified in the present study, namely, WIPI1, BAG1, and PEX3. The STRING database is from the STRING consortium (Swiss Institute for Bioinformatics, CPR NNF Center for Protein Research, EMBL-European Molecular Biology Laboratory).

### 2.8. Statistics

Analysis was performed on 498 human samples, including the selection phase and the first- and second-round validation phases, namely, 45 melanoma and 18 nevi from GEO, 208 melanoma and 147 healthy skin from IST Online, and 47 melanoma and 33 healthy skin from HPA. Within the third-round validation in Oncomine, 31,931 human samples were analyzed from 170 datasets with 22 different cancer types and on 3392 samples from 11 different cancer cell lines. Mean, two-tailed *t* test, Mann-Whitney test, and ROC analysis were carried out, with a threshold *p* value set at ≤0.05. Gaussian distribution was investigated with D'Agostino and Pearson normality test. GraphPad Prism version 5.01 was used (GraphPad Software Inc.).

## 3. Results

### 3.1. ARG Gene Expression Analysis in the GEO Database

**Figure 1:**
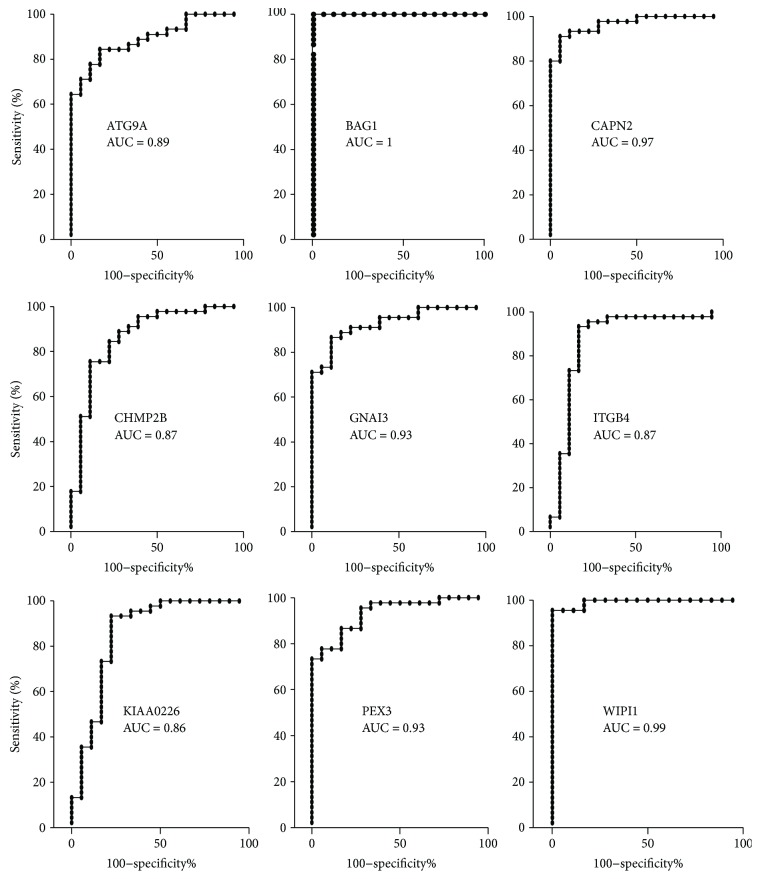
ROC analysis on the expression data of 9 genes never related to melanoma diagnosis or prognosis. The area under the curve (AUC) is plotted as sensitivity% *vs* 100-specificity%. The calculated AUC is reported in each case. The *p* value is <0.0001 in all cases.

Expression data of 222 ARGs (list reported in Supplementary Table 1) were collected from the GDS1375 dataset in GEO. ROC analysis and Mann-Whitney analyses were carried out to measure the ability to discriminate melanoma biopsies from nevi controls. Expression of 42 genes was found to discriminate melanoma biopsies from nevi in a very effective way, i.e., with an AUC ≥ 0.85 and *p* < 0.0001 (Table 1). Most of such 42 genes are known to be involved in melanoma. A Pubmed search carried out on April 19, 2018, identified 9 genes never directly related to melanoma diagnosis. Such 9 genes (namely, ATG9A, BAG1, CAPN2, CHMP2B, GNAI3, ITGB4, KIAA0226, PEX3, and WIPI1) are highlighted in white font on black background in Table 1 and are here considered as novel candidate melanoma markers.  Figure 1 reports the corresponding ROC curve for the expression values; BAG1, PEX3, and WIPI1, selected in the following validation steps, show AUC 1, 0.93, and 0.99, respectively, with extremely high sensitivity and specificity values. Namely, the BAG1 expression level of 1037 shows 100% sensitivity and 100% specificity; the PEX3 expression level of 387.8 shows 77.8% sensitivity and 94.4 specificity; and the WIPI1 expression level of 913.3 shows 95.6% sensitivity and 100% specificity.

### 3.2. First-Round Validation: Gene Expression Analysis in the IST Online Database

**Figure 2:**
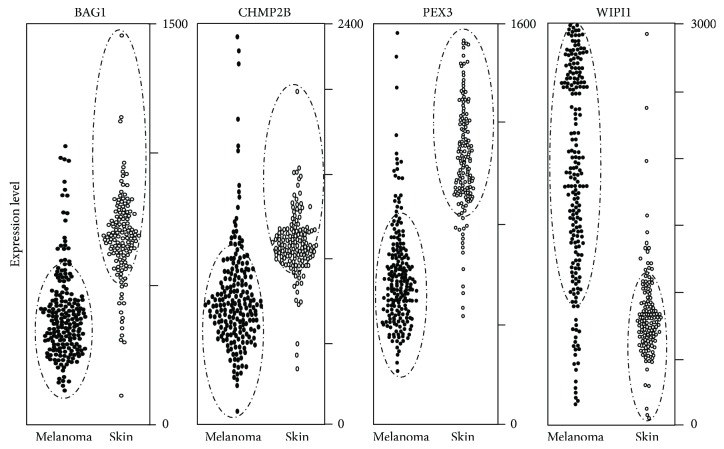
Gene expression according to the IST online database. The four reported genes show different expression levels in melanoma *vs* healthy skin. The expression level of each gene is reported in 208 melanoma biopsies and 147 healthy skin biopsies, according to the IST Online database. Gating indicated with dashed lines include 90% of melanoma and 90% of ctrl skin samples. PEX3, BAG1, and CHMP2B expression in melanoma is clearly lower than healthy skin. WIPI1 expression in melanoma is clearly higher than healthy controls.

The 9 genes reported in Figure 1 were then validated in an independent public database, namely, IST Online, having 208 melanoma patients and 147 healthy skin controls. BAG1, CHMP2B, PEX3, and WIPI1 confirm strongly different expression levels in melanoma *vs* healthy skin in IST Online (Figure 2). Dashed ovals in Figure 2 select 90% of samples from the remaining 10%. We conclude that BAG1, CHMP2B, PEX3, and WIPI1 show a strongly different expression in melanoma *vs* control biopsies, according to data from 418 patients, and have never been related to melanoma according to Pubmed.

### 3.3. Second-Round Validation: Protein Expression Analysis in the Human Protein Atlas Database

**Figure 3:**
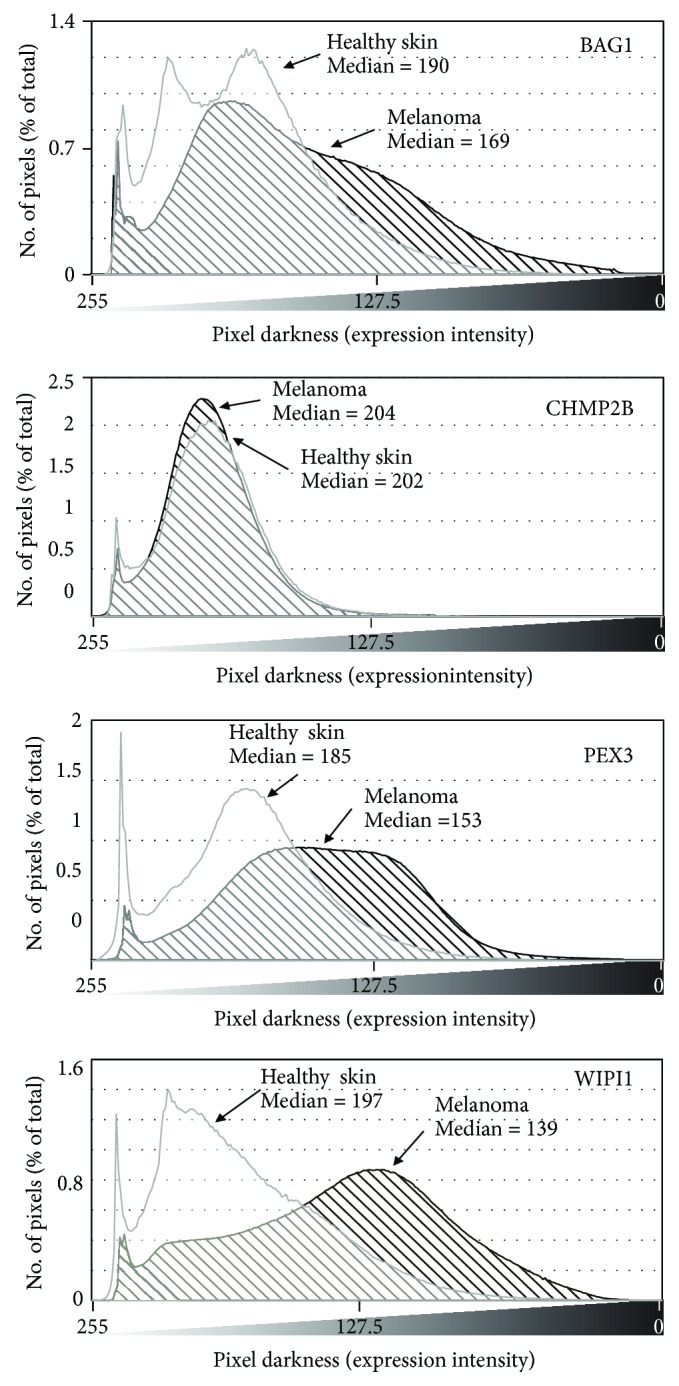
Protein expression according to the Human Protein Atlas. The plot reports the distribution of pixel as function of the expression level. Positions at the right end of the graph indicate higher protein expression. Median level in melanoma samples (dashed curve) shows a clear right shift as compared to healthy skin (gray curve), for BAG1, PEX3, and WIPI1 proteins.

BAG1, CHMP2B, PEX3, and WIPI1 protein expression levels were then analyzed on Human Protein Atlas (HPA) (https://www.proteinatlas.org/). Eighty histological section images from melanoma patients and healthy skin were analyzed. Grayscale conversion and pixel distribution quantification were obtained as reported in Materials and Methods.  Figure 3 shows the distribution plot of the pixels of any given density; the right-end site indicates distribution of darker pixels, i.e., higher expression. Darker pixels are more frequent in melanoma as compared to normal tissues for BAG1, PEX3, and WIPI1, while CHMP2B expression is unchanged. The median pixel-darkness, reported in each panel, indicates the shift toward higher expression of BAG1, PEX3, and WIPI1 in melanoma *vs* healthy skin. Noteworthy, WIPI1 increases in melanoma at both gene (Figure 2) and protein expression levels (Figure 3).

### 3.4. Mechanisms Underlying Functional Interaction of ARGs with Melanoma

#### 3.4.1. Chilibot Analysis

**Figure 4:**
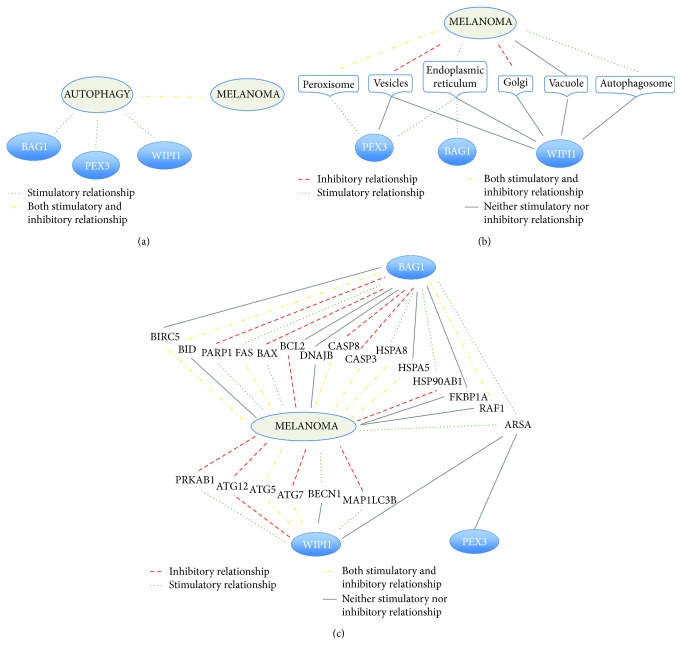
Functional relations reported in Pubmed abstracts, according to Chilibot analysis. Only strong interactive relationships are reported (i.e., interactive relationships reported by at least 5 Pubmed abstracts). Green dotted lines indicate stimulatory relationships; yellow dashed/dotted lines indicate both stimulatory and inhibitory relationships; red dashed lines indicate inhibitory relationships; and continuous gray lines indicate neither stimulatory nor inhibitory relationship, according to Chilibot categories. (a) None of the 3 selected autophagy-related genes has any direct known interactive relationship with melanoma; rather, the relationships are all mediated by autophagy. This indicates that the proposed role of BAG1, PEX3, and WIPI1 in melanoma is novel. (b) Strong interactive relationships of the 3 genes occur with intracellular vesicles, and, through these, they may relate to melanoma. (c) Strong interactive relationships of all 222 ARGs taken from Supplementary Table 1 with melanoma were investigated. Neither BAG1, nor PEX3 nor WIPI1, has a direct strong interactive relationship with melanoma. BAG1 may have indirect strong interactive relationships with melanoma, mediated by BIRC5, BID, PARP1, FAS, BAX, DNAJB, and many others. WIPI1 interaction with melanoma is mediated by PRKAB1, ATG12, ATG5, ATG7, BECN1, and MAP1LC36. Interestingly, ARSA is the only autophagy-related gene having known strong interactive relationships with both BAG1, PEX3, and WIPI1 and with melanoma.

The molecular mechanisms underlying the functional interaction of BAG1, PEX3, and WIPI1 with melanoma were investigated with Chilibot analysis. According to this analysis, no direct and strong relationship is reported so far, of such genes with melanoma, while their interaction to melanoma may be mediated via autophagy (Figure 4(a)) or via several intracellular vesicles (Figure 4(b)) or via several molecules selected from the ARG list (Figure 4(c)). We concluded that the role of BAG1, PEX3, and WIPI1 as potential melanoma markers is novel and their action on melanoma may occur at the intracellular vesicle level via molecular pathways involving other ARGs. Interestingly, ARSA (arylsulfatase A) is the only ARG mediating BAG1, PEX3, and WIPI1 relation with melanoma.

#### 3.4.2. GO Enrichment Analysis

As an additional way to investigate mechanisms underlying the functional interactions of the identified ARGs, the GO enrichment analysis was carried on the 42 genes reported in Table 1. The analysis, available as one of the Gene Ontology tools at http://www.geneontology.org/page/go-enrichment-analysis, identifies GO terms (cellular components, molecular functions, or biological processes) overrepresented or underrepresented in the annotations of the given gene list. A sort of molecular/functional fingerprint of the given gene list is then obtained. The cellular component terms overrepresented in the GO annotations of the given 42-gene list include cytosol, membrane raft, and whole membrane as the most significantly enriched cellular components within the 42-gene list, fully confirming the above reported Chilibot analysis which highlights the role of such genes in intracellular organelle regulation. The same analysis carried out on the molecular function terms indicates protein binding and enzyme binding as the most significantly enriched functions. Finally, the GO enrichment analysis carried out on the biological processes indicates negative regulation of cell death, cellular response to external stimulus, negative regulation of apoptosis, and regulation of cellular response to stress, as the most significantly enriched biological processes.

### 3.5. Prognostic Value

The prognostic value of ARG expression was investigated according to 3-year survival data in 102 melanoma patients reported in Human Protein Atlas, stratified as “high”- or “low”-expressing patients. The prognostic value is calculated by the Human Protein Atlas by classifying patients in two groups on the basis of the FPKM gene expression value. The prognosis of each group is examined by Kaplan-Meier analysis, and the survival outcomes of the two groups are compared by log-rank tests. According to such analysis, BAG1 expression shows a slightly significant prognostic value, i.e., higher survival in low-expressing patients (*p* = 0.04). BAG1 also shows a favorable prognostic value in renal cancer (*p* < 0.0001). PEX3 expression shows no significant prognostic value in melanoma patients while it is a favorable prognostic marker in renal cancer patients (*p* < 0.0001). WIPI1 expression shows a favorable prognostic value in melanoma, with very high statistical significance (*p* = 0.0003).

An additional way to evaluate the potential prognostic values of BAG1, PEX3, and WIPI1 was carried out by assessing their expression in primary *vs* metastatic melanoma samples. The GSE15605 dataset in the GEO database was investigated, which reports expression data from 16 normal skin samples, 46 primary melanoma samples, and 12 metastatic melanoma samples. Expression of all three genes BAG1, PEX3, and WIPI1 was found to be significantly different in normal *vs* primary melanoma samples (*p* < 0.01 in all cases) and normal vs primary + metastatic samples (*p* < 0.001 in all cases), fully confirming the differences observed in the GDS1375 dataset reported in Table 1. BAG1 and PEX3 expression was also found significantly reduced in metastatic *vs* primary melanoma (*p* = 0.00004 and *p* = 0.002, respectively), indicating their possible role in the metastatic dissemination phase.

### 3.6. Third-Round Validation on WIPI1 Overexpression in Melanoma

We then addressed the question of what cancer types show the most significant and relevant WIPI1 upregulation. More than 20 cancer types, several hundred datasets, and several thousand human biopsies and cell line samples were investigated in Oncomine.  Table 2 shows that the WIPI top 5% most relevant overexpression is found in two melanoma datasets, 3 lymphoma, 1 esophagus, and 1 oropharyngeal datasets. When such analysis was carried out on cancer cell lines, mostly melanoma cell lines show a significant and relevant change of WIPI1 (6 melanoma cell lines and 1 myeloma cell line). This data indicate that a strongly significant and relevant WIPI1 overexpression is mostly observed in melanoma and not observed in most other cancer types.

One additional validation was achieved by accessing the Expression Atlas database at EMBL-EBI available at (https://www.ebi.ac.uk/gxa/home). At the homepage of such database, by typing “WIPI1,” “*Homo sapiens*,” and “melanoma” in the search dialog windows, the Pan Cancer Analysis of Whole Genomes Project reports a clear increase of WIPI1 expression in melanoma *vs* normal skin (35 TPM *vs* “below cutoff,” respectively). In addition, according to the Human Protein Atlas, WIPI1 shows in melanoma the highest protein expression, as compared to any other cancer investigated (12 of 12 patients show medium/high WIPI1 protein expression in melanoma, while 9 of 10 in breast cancer and 8 of 10 in prostate cancer and then all others) by HPA007493 antibody staining.

### 3.7. Analysis of the Physical/Functional Features of the Gene Network

**Figure 5:**
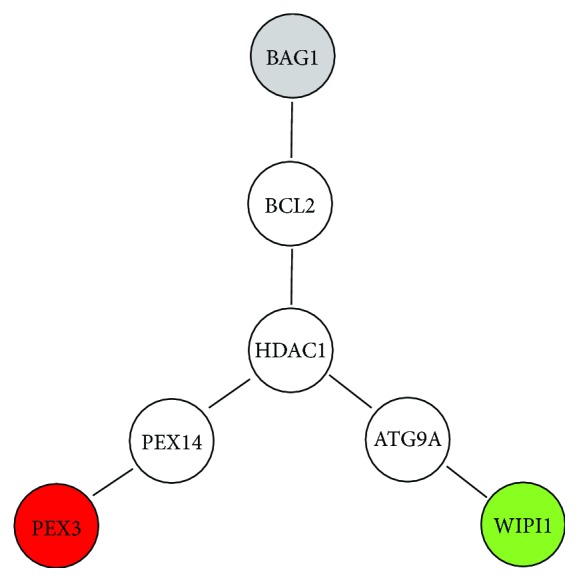
STRING analysis of WIPI1, BAG1, and PEX3 network. BCL2, PEX14, and ATG9A physically/functionally connect BAG1, PEX3, and WIPI1 (respectively) via HDAC1.

STRING analysis allows integrating data regarding protein-protein physical and functional interactions, either predicted or experimentally demonstrated. STRING analysis was carried out to identify intermediate players within the ARGS listed in Supplemental Table 1, able to functionally connect WIPI1 to BAG1 to PEX3. According to this analysis, the tripolar network depicted in Figure 5 was identified, indicating that WIPI1 is physically/functionally related to BAG1 and PEX3 via ATG9A, BCL2, and PEX14, with HDAC1 at the three-arm interconnection. The functional enrichment analysis of this network highlights the following biological processes significantly enriched: “protein targeting to peroxisome,” “cellular response to starvation,” “protein localization to pre-autophagosomal structure” and “nucleophagy.” The enriched “cellular components” are “pre-autophagosomal structure,” “autophagosome membrane,” “peroxisomal membrane,” “whole membrane,” and “autophagosome.”

## 4. Discussion

The current study represents the first extensive analysis of the expression levels of several ARGs in melanoma human biopsies and histological sections. ARG expression was analyzed in 498 human samples from both transcriptomic and proteomic independent databases, according to a multistep validation procedure. BAG1, PEX3, and WIPI1 were identified as the best three novel candidates as validated markers in both DNA and protein melanoma samples, while CHMP2B has validated differential expression at the DNA level, not observed at the protein level. Such genes are known autophagy-related molecules, and their role has been studied in different cancers. BAG1 is a survival promoter in different cancers [25, 26]; WIPI aberrant expression in melanoma cell lines has been reported [27], and its specific mutations are known in melanoma cell lines [28]. Nevertheless, an extensive analysis of their gene and protein expression levels in human samples has not been reported in full manuscripts, yet.

A mechanism underlying the role of these genes as melanoma markers may relate to their physical/functional interactions with intracellular vesicles. Noteworthily, BAG1, PEX3, and WIPI1 are all membrane-associated molecules and intracellular vesicles play a key role in melanoma biology. BAG1 localizes in the nucleus and cytosol; PEX3 in peroxisomes, nucleus, and endoplasmic reticulum; and WIPI1 in cytoskeleton, cytosol, Golgi apparatus, and endosomes. Therefore, proteins coded by these 3 genes interact with such vesicles, as highlighted in Figure 4(b). Intracellular vesicles are emerging as relevant mediators of tumorigenesis. The altered expression of genes/proteins controlling biogenesis and stability of intracellular vesicles may control the melanoma setup. In fact, melanoma-derived exosomes act onto the bone marrow toward melanoma progression and metastasis, carrying the proteome repertoire derived from the primary melanoma [29]. Thus, intracellular vesicles may carry genetic and nongenetic material to “educate” target cells toward a more or less aggressive behavior. Autophagosomes are an additional example of how intracellular vesicle trafficking may control the cellular phenotype. Other intracellular compartments such as melanosomes are connected to the epitope presentation and immune recognition of melanoma-related epitopes [30]. Intracellular vesicle formation in melanoma cells is under GSK3 and Wnt signaling control and directly relates to the melanoma proliferation potential [31, 32]. Formation and maturation of melanosomes and other vesicles are therefore strongly related to autophagy. Melanin synthesis has been shown by us [33] and others [34–37] as one of the key steps in the melanoma setup and progression. Indeed, inhibition of melanogenesis relates to autophagy activation [38], pigmentation enhancement is related to autophagy inhibition [39, 40], and the entire autophagy machinery directly controls melanosome movements and translocations [41]. Further, melanocytes having reduced autophagy undergo senescence and lipid oxidation [6, 42].

BAG1 is a BCL2-associated athanogene; it enhances BCL2 antiapoptotic effects, therefore the reduced expression in melanoma reported in Figure 2 and Table 1 may promote melanoma onset. The HPA database reports higher survival in BAG1 low-expressing melanoma patients (*p* = 0.04) and a favorable prognostic value in renal cancer.

CHMP2B interacts with endosomal originated vesicles, being part of the ESCRT-III complex, which degrades/recycles membrane receptors. This gene is associated with a familial frontotemporal lobar degeneration and with amyotrophic lateral sclerosis. Melanoma association with neurological cancers is known [43], and correlation with other neurological disorders is an emerging field of investigation [44]. Further, CHMP2B has a key role in melanin synthesis or accumulation; in fact, a mutated form of CHMP2B induces melanization in fly eye [45].

PEX3 is involved in biosynthesis and integrity of peroxisome and membrane vesicle assembly. It is reduced in melanoma (Figure 2 and Table 1); within the cancers reported in HPA, the lowest expression is observed in melanoma. As CHMP2B, PEX3 has been related to neurological diseases. Namely, PEX3 mutations are related to Zellweger syndrome (ZWS), a neurologic dysfunction with craniofacial abnormalities and liver dysfunction. According to the “Orphan disease connections” portal (http://csbg.cnb.csic.es/odcs/disease_showresults.php?dis0=Melanoma%20and%20neural%20system%20tumor%20syndrome) [46], melanoma and Zellweger syndrome are considered connected diseases and PEX3 falls in the CDKN2A interactome, one of the most frequently mutated genes in melanoma.

Finally, WIPI1 controls autophagosome assembly and binds phosphoinositides, essential components of any membrane. It is known to have a relevant role in starvation- and calcium-mediated autophagy and in mitophagy. Its expression is strongly increased in melanoma at both DNA and protein levels (Figure 2, Table 1, Figure 3), and it has one of the highest AUC values (Figure 1) and according to HPA shows a favorable prognostic value in melanoma with a very high statistical significance (*p* < 0.0001). WIPI1 is an ATG18 homolog; it colocalizes with the LC3 autophagy marker in melanoma cells [27] and localizes within autophagosomes and endosomes. Via mTOR pathway inhibition, it induces melanogenic enzyme transcription and melanosome formation [47], revealing a specific role of WIPI1 in melanosome maturation. Therefore, WIPI1 and CHMP2B are both involved in melanin metabolism.

MAPK phosphorylation is reduced by WIPI1 knockdown [48], underlying the WIPI1-MAPK axis indicated in Figure 4(c), indicating the possible molecular pathway relating WIPI1 to melanoma setup and progression via MAPK and PD-L1 [49].

Figure 4(c) shows that BAG1, PEX3, and WIPI1 have no known direct relation with melanoma and at least 20 ARGs may mediate their interaction with melanoma. According to Pubmed searches, ARSA, the arylsulfatase A gene, appears to mediate BAG1, PEX3, and WIPI1 interaction with melanoma (Figure 4(c)). Namely, arylsulfatase may control melanoma progression [50]. Its deficiency leads to metachromatic leukodystrophy, a rare genetic lysosomal storage disease with metabolism deficit at the sphingolipid level, affecting membrane structure and function, pointing again toward membrane metabolism. The STRING analysis carried out on ARSA indicates the most significant biological process enrichment in the ARSA network as the “glycosphingolipid metabolic process” (false discovery rate 1.25*E* − 21), the most significant molecular function enrichment as the “sulfuric ester hydrolase activity” (false discovery rate 8.8*E* − 17), and the most significant cellular component enrichment as the “endoplasmic reticulum lumen” (false discovery rate 3.9*E* − 14), highlighting again a biochemical modification at the lipid level within intracellular vesicles. Finally, regarding the tripolar connection reported in Figure 5, it should be noticed that HDAC1, predicted by the STRING analysis as the central player physically/functionally connecting WIPI1 to BAG1 to PEX3, has been shown to be involved in melanoma drug resistance and melanoma progression [51–53]. STRING analysis carried out on other secondary hits reported in Table 1 indicates functional enrichment of axon guidance and phagocytosis (for CAPN2), cell separation and viral budding (for CHMP2B), response to glucagon and regulation of G-protein-coupled receptors (for GNAI3), hemidesmosome assembly (for ITGB4), endosomal transport (for KIAA0226), and unfolded protein response (for EIF2AK3). In all such cases, cellular component enrichment analysis identifies cellular membrane components.

In conclusion, the present study highlights 3 genes (BAG1, PEX3, and WIPI1), known to play a key role in autophagy, as novel relevant melanoma markers. WIPI1 is significantly upregulated at both gene and protein levels in melanoma samples, showing the highest expression fold change, the highest ability to discriminate healthy individuals from patients, and the strongest prognostic value, and it has never been related to melanoma. Therefore, such molecules may represent valuable novel markers of melanoma setup and progression.

## Figures and Tables

**Table 1:** ROC analysis of ARGs, according to the GDS1375 dataset from GEO database. Only genes showing AUC ≥ 0.85 are reported; genes highlighted in white font on black background have still unknown relationships with melanoma diagnosis or prognosis. CTSB shows the highest fold increase (10-fold increase in melanoma *vs* nevi); WIPI1 shows the second highest fold increase (8.1-fold increase). EGFR shows the highest fold decrease (−10.7 fold), PTK6 the second highest fold decrease (−8.8 fold).

	Symbol	Mean expression in melanoma	Mean expression in nevi	Fold change melanoma vs nevi	AUC	*p* value of the computed AUC	Number of Pubmed abstracts with the gene symbol and “melanoma” in ALL field (up to April 19 2018)
1	ATF4	5611	7598	+1.3	0.87	<0.0001	≥1
2	ATG4B	1016	739	+1.4	0.88	<0.0001	≥1
3	**ATG9A**	**1014**	**735**	**+1.4**	**0.89**	**<0.0001**	**0**
4	**BAG1**	**727**	**1891**	**−2.6**	**1**	**<0.0001**	**0**
5	BAG3	1098	1831	−1.7	0.87	<0.0001	≥1
6	BAX	498	186	+2.7	0.93	<0.0001	≥1
7	BCL2	1409	180	+7.8	0.99	<0.0001	≥1
8	BCL2L1	1244	375	+3.3	0.99	<0.0001	≥1
9	BIRC5	590	230	+2.6	0.92	<0.0001	>1
10	**CAPN2**	**9236**	**5096**	**+1.8**	**0.96**	**<0.0001**	**0**
11	CAPNS1	8826	4392	+2.0	0.93	<0.0001	≥1
12	CDKN1A	2790	1397	+2.0	0.90	<0.0001	≥1
13	CDKN2A	650	335	+1.9	0.86	<0.0001	≥1
14	CFLAR	472	796	−1.7	0.85	<0.0001	≥1
15	**CHMP2B**	**282**	**561**	**−2.0**	**0.87**	**<0.0001**	**0**
16	CTSB	16,713	1655	+10.0	0.99	<0.0001	≥1
17	CTSD	2115	1029	+2.0	0.89	<0.0001	≥1
18	CX3CL1	266	627	−2.3	0.91	<0.0001	≥1
19	EGFR	184	1976	−10.7	0.98	<0.0001	≥1
20	EIF2AK3	566	282	+2.0	0.93	<0.0001	≥1
21	EIF2S1	16,903	9247	+1.8	0.90	<0.0001	0
22	ERBB2	2107	1695	+1.2	0.90	<0.0001	≥1
23	FAS	338	681	−2.0	0.89	<0.0001	≥1
24	FOXO1	482	1055	−2.2	0.96	<0.0001	≥1
25	**GNAI3**	**193**	**362**	**−1.9**	**0.94**	**<0.0001**	**0**
26	HDAC1	1614	1146	+1.4	0.86	<0.0001	≥1
27	HSPA5	3830	2390	+1.6	0.86	<0.0001	≥1
28	HSPB8	200	947	−4.7	0.94	<0.0001	≥1
29	ITGA3	2436	497	+4.9	0.95	<0.0001	≥1
30	**ITGB4**	**206**	**944**	**−4.6**	**0.87**	**<0.0001**	**0**
31	**KIAA0226**	**186**	**104**	**+1.8**	**0.85**	**<0.0001**	**0**
32	MAPK1	730	1339	−1.8	0.86	<0.0001	≥1
33	MLST8	833	453	+1.8	0.90	<0.0001	≥1
34	NFE2L2	1410	2622	−1.8	0.91	<0.0001	≥1
35	PARP1	2212	975	+2.3	0.99	<0.0001	≥1
36	PEA15	5307	3477	+1.5	0.94	<0.0001	≥1
37	**PEX3**	**343**	**670**	**−1.9**	**0.93**	**<0.0001**	**0**
38	PTK6	63	556	−8.8	0.96	<0.0001	≥1
39	SQSTM1	4197	2636	+1.6	0.95	<0.0001	≥1
40	TP63	131	1067	−8.1	0.93	<0.0001	≥1
41	TP73	578	785	−1.3	0.89	<0.0001	≥1
42	**WIPI1**	**3043**	**374**	**+8.1**	**0.99**	<**0.0001**	**0**

**Table 2:** Cancer types showing significant overexpression of WIPI1. In human biopsy cancers (left column), 2 melanoma datasets are within the top 5% ranked datasets with relevant WIPI1 overexpression. In human cell lines (right column), 6 melanoma and 1 myeloma cell lines are present in the top 5% ranked datasets of cell lines with relevant WIPI1 overexpression. Left column: WIPI1 expression in 31,931 human biopsy samples from more than 20 different cancer types was analyzed, namely, biopsies from bladder; brain and central nervous systems; and breast, colorectal, cervical, esophageal, gastric, head and neck, kidney, leukemia, liver, lung, lymphoma, melanoma, myeloma, ovarian, pancreatic, prostate, sarcoma, mesothelioma, and seminoma cancers. Right column: 26 different datasets with 7410 samples from 18 different cancer types were analyzed (melanoma, lymphoma, leukemia, testicular germ cell neoplasm, breast, brain, liver, gastrointestinal, sarcoma, lung, prostate, colorectal, kidney, ovarian, bladder, pancreatic, and esophageal tumors). The following stringent thresholds were selected: *p* ≤ 0.0001; fold change (FC) ≥ 3; gene rank top 5% (data from Oncomine, http://www.oncomine.com).

Top 5% human biopsies of different cancer types, with WIPI1 overexpression	Top 5% human cancer cell lines, with WIPI1 overexpression
Riker melanoma dataset: Cutaneous melanoma: *p* = 0.00002; FC 3.1; rank 79	Shankavaram melanoma cell line: *p* = 0.00001; FC 6.8; rank 207
Compagno lymphoma dataset: Diffuse large B-cell lymphoma: *p* = 4.5*E* − 27; FC 4.9; rank 111	Compendia melanoma cell line: *p* = 0.00001; FC 6.7; rank 218
Compagno lymphoma dataset: Activated B-cell like diffuse large B-cell lymphoma: *p* = 1.6*E* − 10; FC 4.2; rank 544	Garnett melanoma cell line: *p* = 2.2*E* − 13; FC 5.4; rank 232
Hao esophagus dataset: esophageal adenocarcinoma: *p* = 1.6*E* − 10; FC 4.2; rank 544	Adai melanoma cell line: *p* = 3.4*E* − 8; FC 7.4; rank 285
Piccaluga lymphoma: Anaplastic large cell lymphoma *p* = 2.2*E* − 6; FC 4.9; rank 193	Barretina melanoma cell line: *p* = 1.5*E* − 19; FC 4.77; rank 301
Pyeon multicancer dataset: oropharyngeal carcinoma *p* = 2.3*E* − 5, FC 3.5; rank 323	Barretina myeloma cell line: *p* = 1.2*E* − 8; FC 3.3; rank 551
Talantov melanoma dataset: cutaneous melanoma: *p* = 7.3*E* − 7; FC 8.4; rank 467	Wagner melanoma cell line: *p* = 4.8*E* − 8; FC 5.2; rank 533

## Data Availability

The data used to support the findings of this study are available from the corresponding author upon request.

## Supplementary Materials

Supplementary Materials Supplementary Table 1: autophagy-related genes (ARGs) investigated in the present study.
