# Supplementary material for: WIPI1, BAG1, and PEX3 Autophagy-Related Genes Are Relevant Melanoma Markers
Source: Oxid Med Cell Longev. 2018 Dec 2;2018:1471682. doi: 10.1155/2018/1471682 (PMC6304818; doi:10.1155/2018/1471682)
Supplement: Supplementary Materials — Supplementary Table 1: autophagy-related genes (ARGs) investigated in the present study. [file 1471682.f1.docx]

**Supplementary Table 1**

**Autophagy Related genes (ARGs) investigated in the present study**.

|  | **Name** | **Symbol** |
| --- | --- | --- |
| 1 | Autophagy/beclin-1 regulator 1 | AMBRA1 |
| 2 | Apolipoprotein L, 1 | APOL1 |
| 3 | Aryl hydrocarbon receptor nuclear translocator | ARNT |
| 4 | Aarylsulfatase A | ARSA |
| 5 | Aarylsulfatase B | ARSB |
| 6 | Aactivating transcription factor 4 | ATF4 |
| 7 | Activating transcription factor 6 | ATF6 |
| 8 | ATG10 autophagy related 10 homolog (S. cerevisiae) | ATG10 |
| 9 | ATG12 autophagy related 12 homolog (S. cerevisiae) | ATG12 |
| 10 | ATG16 autophagy related 16-like 1 (S. cerevisiae) | ATG16L1 |
| 11 | ATG16 autophagy related 16-like 2 (S. cerevisiae) | ATG16L2 |
| 12 | ATG2 autophagy related 2 homolog A (S. cerevisiae) | ATG2A |
| 13 | ATG2 autophagy related 2 homolog B (S. cerevisiae) | ATG2B |
| 14 | ATG3 autophagy related 3 homolog (S. cerevisiae) | ATG3 |
| 15 | ATG4 autophagy related 4 homolog A (S. cerevisiae) | ATG4A |
| 16 | ATG4 autophagy related 4 homolog B (S. cerevisiae) | ATG4B |
| 17 | ATG4 autophagy related 4 homolog C (S. cerevisiae) | ATG4C |
| 18 | ATG4 autophagy related 4 homolog D (S. cerevisiae) | ATG4D |
| 19 | ATG5 autophagy related 5 homolog (S. cerevisiae) | ATG5 |
| 20 | ATG7 autophagy related 7 homolog (S. cerevisiae) | ATG7 |
| 21 | ATG9 autophagy related 9 homolog A (S. cerevisiae) | ATG9A |
| 22 | ATG9 autophagy related 9 homolog B (S. cerevisiae) | ATG9B |
| 23 | 5aminoimidazole4carboxamide ribonucleotide formyltransferase/IMP cyclohydrolase | ATIC |
| 24 | BCL2-associated athanogene | BAG1 |
| 25 | BCL2-associated athanogene 3 | BAG3 |
| 26 | BCL2-antagonist/killer 1 | BAK1 |
| 27 | BCL2-associated X protein | BAX |
| 28 | B-cell CLL/lymphoma 2 | BCL2 |
| 29 | BCL2-like 1 | BCL2L1 |
| 30 | Beclin 1, autophagy related | BECN1 |
| 31 | BH3 interacting domain death agonist | BID |
| 32 | baculoviral IAP repeat-containing 5 | BIRC5 |
| 33 | baculoviral IAP repeat-containing 6 | BIRC6 |
| 34 | BCL2/adenovirus E1B 19kDa interacting protein 1 | BNIP1 |
| 35 | BCL2/adenovirus E1B 19kDa interacting protein 3 | BNIP3 |
| 36 | BCL2/adenovirus E1B 19kDa interacting protein 3-like | BNIP3L |
| 37 | Chromosome 12 open reading frame 44 | C12orf44 |
| 38 | Chromosome 17 open reading frame 88 | C17orf88 |
| 39 | Calcium binding and coiled-coil domain 2 | CALCOCO2 |
| 40 | Calcium/calmodulin-dependent protein kinase kinase 2, beta | CAMKK2 |
| 41 | Calnexin | CANX |
| 42 | Calpain 1, (mu/I) large subunit | CAPN1 |
| 43 | Calpain 10 | CAPN10 |
| 44 | Calpain 2, (m/II) large subunit | CAPN2 |
| 45 | Calpain, small subunit 1 | CAPNS1 |
| 46 | Caspase 1, apoptosis-related cysteine peptidase (interleukin 1, beta, convertase) | CASP1 |
| 47 | Caspase 3, apoptosis-related cysteine peptidase | CASP3 |
| 48 | Caspase 4, apoptosis-related cysteine peptidase | CASP4 |
| 49 | Caspase 8, apoptosis-related cysteine peptidase | CASP8 |
| 50 | Chemokine (C-C motif) ligand 2 | CCL2 |
| 51 | Chemokine (C-C motif) receptor 2 | CCR2 |
| 52 | CD46 molecule, complement regulatory protein | CD46 |
| 53 | Cyclin-dependent kinase inhibitor 1A (p21, Cip1) | CDKN1A |
| 54 | Cyclin-dependent kinase inhibitor 1B (p27, Kip1) | CDKN1B |
| 55 | Cyclin-dependent kinase inhibitor 2A (melanoma, p16, inhibits CDK4) | CDKN2A |
| 56 | CASP8 and FADD-like apoptosis regulator | CFLAR |
| 57 | Chromatin modifying protein 2B | CHMP2B |
| 58 | Chromatin modifying protein 4B | CHMP4B |
| 59 | Ceroid-lipofuscinosis, neuronal 3 | CLN3 |
| 60 | Cathepsin B | CTSB |
| 61 | Cathepsin D | CTSD |
| 62 | Cathepsin L1 | CTSL1 |
| 63 | Chemokine (C-X3-C motif) ligand 1 | CX3CL1 |
| 64 | Chemokine (C-X-C motif) receptor 4 | CXCR4 |
| 65 | Death-associated protein kinase 1 | DAPK1 |
| 66 | Death-associated protein kinase 2 | DAPK2 |
| 67 | DNA-damage-inducible transcript 3 | DDIT3 |
| 68 | DIRAS family, GTP-binding RAS-like 3 | DIRAS3 |
| 69 | Deleted in liver cancer 1 | DLC1 |
| 70 | DnaJ (Hsp40) homolog, subfamily B, member 1 | DNAJB1 |
| 71 | DnaJ (Hsp40) homolog, subfamily B, member 9 | DNAJB9 |
| 72 | DNA-damage regulated autophagy modulator 1 | DRAM1 |
| 73 | ER degradation enhancer, mannosidase alpha-like 1 | EDEM1 |
| 74 | Eukaryotic translation elongation factor 2 | EEF2 |
| 75 | Eukaryotic elongation factor-2 kinase | EEF2K |
| 76 | Epidermal growth factor receptor | EGFR |
| 77 | Eukaryotic translation initiation factor 2-alpha kinase 2 | EIF2AK2 |
| 78 | Eukaryotic translation initiation factor 2-alpha kinase 3 | EIF2AK3 |
| 79 | Eukaryotic translation initiation factor 2, subunit 1 alpha, 35kDa | EIF2S1 |
| 80 | Eukaryotic translation initiation factor 4E binding protein 1 | EIF4EBP1 |
| 81 | Eeukaryotic translation initiation factor 4 gamma, 1 | EIF4G1 |
| 82 | v-Erb-b2 erythroblastic leukemia viral oncogene homolog 2, neuro/glioblastoma derived oncogene homolog (avian) | ERBB2 |
| 83 | Endoplasmic reticulum to nucleus signaling 1 | ERN1 |
| 84 | ERO1-like (S. cerevisiae) | ERO1L |
| 85 | Fas (TNFRSF6)-associated via death domain | FADD |
| 86 | Family with sequence similarity 48, member A | FAM48A |
| 87 | Fas (TNF receptor superfamily, member 6) | FAS |
| 88 | FK506 binding protein 1A, 12kDa | FKBP1A |
| 89 | FK506 binding protein 1B, 12.6 kDa | FKBP1B |
| 90 | FBJ murine osteosarcoma viral oncogene homolog | FOS |
| 91 | Forkhead box O1 | FOXO1 |
| 92 | Forkhead box O3 | FOXO3 |
| 93 | Glucosidase, alpha; acid | GAA |
| 94 | GABA(A) receptor-associated protein | GABARAP |
| 95 | GABA(A) receptor-associated protein like 1 | GABARAPL1 |
| 96 | GABA(A) receptor-associated protein-like 2 | GABARAPL2 |
| 97 | Glyceraldehyde-3-phosphate dehydrogenase | GAPDH |
| 98 | Guanine nucleotide binding protein (G protein) alpha inhibiting activity polypeptide 3 | GNAI3 |
| 99 | Guanine nucleotide binding protein (G protein), beta polypeptide 2-like 1 | GNB2L1 |
| 100 | Golgi-associated PDZ and coiled-coil motif containing | GOPC |
| 101 | Glutamate receptor, ionotropic, delta 1 | GRID1 |
| 102 | Glutamate receptor, ionotropic, delta 2 | GRID2 |
| 103 | Histone deacetylase 1 | HDAC1 |
| 104 | Histone deacetylase 6 | HDAC6 |
| 105 | Hepatocyte growth factor-regulated tyrosine kinase substrate | HGS |
| 106 | Hypoxia inducible factor 1, alpha subunit (basic helix-loop-helix transcription factor) | HIF1A |
| 107 | Heat shock protein 90kDa alpha (cytosolic), class B member 1 | HSP90AB1 |
| 108 | Heat shock 70kDa protein 5 (glucose-regulated protein, 78kDa) | HSPA5 |
| 109 | Heat shock 70kDa protein 8 | HSPA8 |
| 110 | Heat shock 22kDa protein 8 | HSPB8 |
| 111 | Interferon, gamma | IFNG |
| 112 | Inhibitor of kappa light polypeptide gene enhancer in B-cells, kinase beta | IKBKB |
| 113 | Inhibitor of kappa light polypeptide gene enhancer in B-cells, kinase epsilon | IKBKE |
| 114 | Interleukin 24 | IL24 |
| 115 | Immunity-related GTPase family, M | IRGM |
| 116 | Integrin, alpha 3 (antigen CD49C, alpha 3 subunit of VLA-3 receptor) | ITGA3 |
| 117 | Integrin, alpha 6 | ITGA6 |
| 118 | Integrin, beta 1 (fibronectin receptor, beta polypeptide, antigen CD29 includes MDF2, MSK12) | ITGB1 |
| 119 | Integrin, beta 4 | ITGB4 |
| 120 | Inositol 1,4,5-triphosphate receptor, type 1 | ITPR1 |
| 121 | KIAA0226 | KIAA0226 |
| 122 | KIAA0652 | KIAA0652 |
| 123 | KIAA0831 | KIAA0831 |
| 124 | Kinesin family member 5B | KIF5B |
| 125 | Kelch-like 24 (Drosophila) | KLHL24 |
| 126 | Lysosomal-associated membrane protein 1 | LAMP1 |
| 127 | Lysosomal-associated membrane protein 2 | LAMP2 |
| 128 | Microtubule-associated protein 1 light chain 3 alpha | MAP1LC3A |
| 128 | Microtubule-associated protein 1 light chain 3 beta | MAP1LC3B |
| 130 | Microtubule-associated protein 1 light chain 3 gamma | MAP1LC3C |
| 131 | Mitogen-activated protein kinase kinase 7 | MAP2K7 |
| 132 | Mitogen-activated protein kinase 1 | MAPK1 |
| 133 | Mitogen-activated protein kinase 3 | MAPK3 |
| 134 | Mitogen-activated protein kinase 8 | MAPK8 |
| 135 | Mitogen-activated protein kinase 8 interacting protein 1 | MAPK8IP1 |
| 136 | Mitogen-activated protein kinase 9 | MAPK9 |
| 137 | Membrane-bound transcription factor peptidase, site 2 | MBTPS2 |
| 138 | MTOR associated protein, LST8 homolog (S. cerevisiae) | MLST8 |
| 139 | Myotubularin related protein 14 | MTMR14 |
| 140 | Mechanistic target of rapamycin (serine/threonine kinase) | MTOR |
| 141 | v-myc myelocytomatosis viral oncogene homolog (avian) | MYC |
| 142 | Nuclear assembly factor 1 homolog (S. cerevisiae) | NAF1 |
| 143 | Nicotinamide phosphoribosyltransferase | NAMPT |
| 144 | Neighbor of BRCA1 gene 1 | NBR1 |
| 145 | NCK-associated protein 1 | NCKAP1 |
| 146 | Nuclear factor (erythroid-derived 2)-like 2 | NFE2L2 |
| 147 | Nuclear factor of k light polypeptide gene enhancer in B-cells 1 | NFKB1 |
| 148 | NK2 transcription factor related, locus 3 (Drosophila) | NKX2-3 |
| 149 | NLR family, CARD domain containing 4 | NLRC4 |
| 150 | Niemann-Pick disease, type C1 | NPC1 |
| 151 | Neuregulin 1 | NRG1 |
| 152 | Neuregulin 2 | NRG2 |
| 153 | Neuregulin 3 | NRG3 |
| 154 | Prolyl 4-hydroxylase, beta polypeptide | P4HB |
| 155 | Parkinson disease (autosomal recessive, juvenile) 2, parkin | PARK2 |
| 156 | Poly (ADP-ribose) polymerase 1 | PARP1 |
| 157 | Phosphoprotein enriched in astrocytes 15 | PEA15 |
| 158 | Proline, glutamate and leucine rich protein 1 | PELP1 |
| 159 | Peroxisomal biogenesis factor 14 | PEX14 |
| 160 | Peroxisomal biogenesis factor 3 | PEX3 |
| 161 | Phosphoinositide-3-kinase, class 3 | PIK3C3 |
| 162 | Phosphoinositide-3-kinase, regulatory subunit 4 | PIK3R4 |
| 163 | PTEN induced putative kinase 1 | PINK1 |
| 164 | Protein phosphatase 1, regulatory (inhibitor) subunit 15A | PPP1R15A |
| 165 | Protein kinase, AMP-activated, beta 1 non-catalytic subunit | PRKAB1 |
| 166 | Protein kinase, cAMP-dependent, regulatory, type I, alpha | PRKAR1A |
| 167 | Protein kinase C, delta | PRKCD |
| 168 | Protein kinase C, theta | PRKCQ |
| 169 | Phosphatase and tensin homolog | PTEN |
| 170 | PTK6 protein tyrosine kinase 6 | PTK6 |
| 171 | RAB11A, member RAS oncogene family | RAB11A |
| 172 | RAB1A, member RAS oncogene family | RAB1A |
| 173 | RAB24, member RAS oncogene family | RAB24 |
| 174 | RAB33B, member RAS oncogene family | RAB33B |
| 175 | RAB5A, member RAS oncogene family | RAB5A |
| 176 | RAB7A, member RAS oncogene family | RAB7A |
| 177 | Ras-related C3 botulinum toxin substrate 1 (rho family small GTP bind. protein Rac1) | RAC1 |
| 178 | v-Raf-1 murine leukemia viral oncogene homolog 1 | RAF1 |
| 179 | Retinoblastoma 1 | RB1 |
| 180 | RB1-inducible coiled-coil 1 | RB1CC1 |
| 181 | v-Rel reticuloendotheliosis viral oncogene homolog A (avian) | RELA |
| 182 | Regulator of G-protein signaling 19 | RGS19 |
| 183 | Ras homolog enriched in brain | RHEB |
| 184 | Ribosomal protein S6 kinase, 70kDa, polypeptide 1 | RPS6KB1 |
| 185 | Regulatory associated protein of MTOR, complex 1 | RPTOR |
| 186 | SAR1 homolog A (S. cerevisiae) | SAR1A |
| 187 | Serpin peptidase inhibitor, clade A (alpha-1 antiproteinase, antitrypsin), member 1 | SERPINA1 |
| 188 | Sestrin 2 | SESN2 |
| 189 | SH3-domain GRB2-like endophilin B1 | SH3GLB1 |
| 190 | Sirtuin (silent mating type information regulation 2 homolog) 1 (S. cerevisiae) | SIRT1 |
| 191 | Sirtuin (silent mating type information regulation 2 homolog) 2 (S. cerevisiae) | SIRT2 |
| 192 | Sphingosine kinase 1 | SPHK1 |
| 193 | Spinster homolog 1 (Drosophila) | SPNS1 |
| 194 | Sequestosome 1 | SQSTM1 |
| 195 | Ssuppression of tumorigenicity 13 (colon carcinoma) | ST13 |
| 196 | Serine/threonine kinase 11 | STK11 |
| 197 | TANK-binding kinase 1 | TBK1 |
| 198 | Transmembrane 9 superfamily member 1 | TM9SF1 |
| 199 | Transmembrane protein 49 | TMEM49 |
| 200 | Transmembrane protein 74 | TMEM74 |
| 201 | Tumor necrosis factor (ligand) superfamily, member 10 | TNFSF10 |
| 202 | Tumor protein p53 | TP53 |
| 203 | Tumor protein p53 inducible nuclear protein 2 | TP53INP2 |
| 204 | Tumor protein p63 | TP63 |
| 205 | Tumor protein p73 | TP73 |
| 206 | Tuberous sclerosis 1 | TSC1 |
| 207 | Tuberous sclerosis 2 | TSC2 |
| 208 | Tumor suppressor candidate 1 | TUSC1 |
| 209 | unc-51-like kinase 1 (C. elegans) | ULK1 |
| 210 | unc-51-like kinase 2 (C. elegans) | ULK2 |
| 211 | unc-51-like kinase 3 (C. elegans) | ULK3 |
| 212 | Ubiquitin specific peptidase 10 | USP10 |
| 213 | UV radiation resistance associated gene | UVRAG |
| 214 | Vesicle-associated membrane protein 3 (cellubrevin) | VAMP3 |
| 215 | Vesicle-associated membrane protein 7 | VAMP7 |
| 216 | Vascular endothelial growth factor A | VEGFA |
| 217 | WD repeat and FYVE domain containing 3 | WDFY3 |
| 218 | WD repeat domain 45 | WDR45 |
| 219 | WDR45-like | WDR45L |
| 220 | WD repeat domain, phosphoinositide interacting 1 | WIPI1 |
| 221 | WD repeat domain, phosphoinositide interacting 2 | WIPI2 |
| 222 | Zinc finger, FYVE domain containing 1 | ZFYVE1 |
